# Presence of mismatches between diagnostic PCR assays and coronavirus SARS-CoV-2 genome

**DOI:** 10.1098/rsos.200636

**Published:** 2020-06-10

**Authors:** Kashif Aziz Khan, Peter Cheung

**Affiliations:** Department of Biology, York University, 4700 Keele Street, Toronto, Canada M3 J 1P3

**Keywords:** COVID-19, coronavirus SARS-CoV-2, diagnosis, sequence variation, polymerase chain reaction (PCR), primer–template mismatch

## Abstract

Severe acute respiratory syndrome coronavirus 2 (SARS-CoV-2; initially named as 2019-nCoV) is responsible for the recent COVID-19 pandemic and polymerase chain reaction (PCR) is the current standard method for its diagnosis from patient samples. This study conducted a reassessment of published diagnostic PCR assays, including those recommended by the World Health Organization (WHO), through the evaluation of mismatches with publicly available viral sequences. An exhaustive evaluation of the sequence variability within the primer/probe target regions of the viral genome was performed using more than 17 000 viral sequences from around the world. The analysis showed the presence of mutations/mismatches in primer/probe binding regions of 7 assays out of 27 assays studied. A comprehensive bioinformatics approach for *in silico* inclusivity evaluation of PCR diagnostic assays of SARS-CoV-2 was validated using freely available software programs that can be applied to any diagnostic assay of choice. These findings provide potentially important information for clinicians, laboratory professionals and policy-makers.

## 1. Introduction

On 31 December 2019, a cluster of 41 pneumonia cases of unknown aetiology in Wuhan, China, were reported to the World Health Organization (WHO). Subsequently, a novel coronavirus of zoonotic origin, severe acute respiratory syndrome coronavirus 2 (SARS-CoV-2; initially named as 2019-nCoV), was isolated from the patients [1–3]. The virus has spread to more than 200 countries and territories resulting in global coronavirus disease 2019 (COVID-19) pandemic [4]. The rapid spread of the virus is partially attributed to the transmission by asymptomatic carriers or mildly symptomatic cases [5,6]. Early diagnostic testing is an important tool for policy-makers to make public health decisions to contain the outbreak.

The virus from the patients was identified and sequenced early in the outbreak [1,7] and resulted in the development of several polymerase chain reaction (PCR) detection protocols by multiple national organizations that were published by the WHO [8]. In addition, several other methods have been developed and published in the literature recently [5,7,9–15]. However, the molecular diagnosis of SARS-CoV-2 may be jeopardized by potential preanalytical and analytical vulnerabilities including lack of harmonization of primers and probes [16]. Given the potential for the viruses to mutate, genetic variations in the viral genome at primer/probe binding regions can result in potential mismatches and false-negative results [17]. For example, primer and template mismatches have been reported to impede proper diagnosis of several viruses including influenza virus [18–21], respiratory syncytial virus [22], dengue virus [23], rabies virus [24], human immunodeficiency virus-1 [25,26] and hepatitis B virus [27,28].

SARS-CoV-2 is an enveloped positive-strand RNA virus classified as a member of family *Coronaviridae* in the genus *Betacoronavirus* along with SARS-CoV and Middle East respiratory syndrome (MERS)-CoV [29]. The sequence analysis of SARS-CoV-2 isolates has shown that its single-stranded RNA genome is approximately 30 kb in size [1,7,30]. Based on similarity with SARS-CoV, SARS-CoV-2 genome has been predicted to encode at least 10 open reading frames (ORFs) for structural and accessory proteins. As per current annotation (NC_045512.2), these viral ORFs encode replicase ORF1ab, spike (S), envelope (E), membrane (M) and nucleocapsid (N), and at least six accessory proteins (3a, 6, 7a, 7b, 8 and 10) [31].

Human coronaviruses encode a proofreading exoribonuclease, nsp14-ExoN, for maintaining replication fidelity and thus have a relatively slower mutation rate than other RNA viruses [32,33]. SARS-CoV-2 encodes nsp14-ExoN as well [1], but mutations have been described in the genome for circulating SARS-CoV-2 [34–38]. Some laboratories have performed the alignment of diagnostic primers/probes with a limited number of viral sequences and have reported some mismatches [39,40] which may lead to false-negative results [41]. The use of several commercially developed diagnostic assays has also been permitted around the world with limited regulatory approval due to the pandemic emergency [42]. However, the limit of detection of these assays differs considerably and can also lead to false-negative results [43]. As there are already reports of false-negative diagnosis of COVID-19 [44–48], there is a need for verification of potential primer/probe mismatch with the sequences of viral isolates being isolated from around the world. The American Society for Microbiology COVID-19 International Summit held on 23 March 2020 recommended routine verification of sequence mutations in primer and probe binding regions of the viral genome for optimal virus detection [49].

The objective of this study is the *in silico* reassessment of previously published PCR primers/probes for COVID-19 diagnosis. This was performed through the evaluation of the sequence variability within the primer/probe target regions of SARS-CoV-2 viral isolates from around the world. The absence of any mutations and mismatches in target regions of the assay used would provide a higher degree of confidence in the test results obtained while the presence of mutations could help guide the strategies for the reassessment of diagnostic assays. We believe that these findings provide potentially important information for clinicians, laboratory professionals and policy-makers.

## 2. Methods

**Figure 1.**
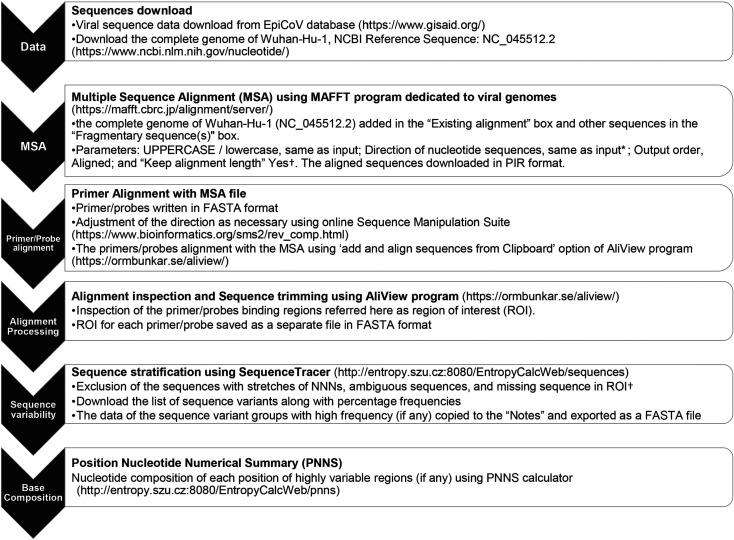
Sequence tracing pipeline used in the study. *The direction can be adjusted by selecting the option ‘Adjust direction according to the first sequence’, if needed. ^†^The change was made with editorial approval after Stage 1.

This study was pre-registered on the Open Science Framework (OSF); the accepted Stage 1 registration can be viewed at (https://osf.io/ym8gc). Minor deviations from protocol are identified in footnotes. The study design planner is included in table 1. The summary of the sequence tracing pipeline is shown in figure 1.

**Table 1.** Study design planner.

question	hypothesis	sampling plan (e.g. power analysis)	analysis plan	interpretation given different outcomes	obtained results and interpretation
are there any mutations in the primer/probe binding regions of the SARS-CoV-2 genome for PCR assays published in the literature?	as the virus can potentially mutate during the outbreak, mutations in the primer/probe binding regions can result in mismatches with primer/probe template	17 026 viral isolates would be downloaded from GISAID EpiCov database *inclusion criteria:* only full length (>29 000 bp) *exclusion criteria:* the sequences with stretches of NNNs, ambiguous sequences, and missing sequences in the region of interest (ROI) will be considered low quality and would be excluded	sequences would be aligned using MAFFT low-quality sequences would be excluded from the alignment and sequence variability would be traced *in silico* using SequenceTracer the highly variable region, if any, would be further analysed for nucleotide composition at each position using positional nucleotide numerical summary (PNNS) the complete genome of Wuhan-Hu-1 from the National Center for Biotechnology Information (NCBI) would act as a positive control (NCBI Reference Sequence: NC_045512.2)	in the event of a negative result, it would be concluded that there is no evidence of a difference between primer/probe and viral isolates this would serve as a reference for researchers and laboratory professionals using PCR assays for the detection of SARS-CoV-2	the analysis showed the presence of mismatches/mutations in primer/probe binding regions of 7 assays out of 27 assays studied

### 2.1. Selection of primers and probes

A total of 27 PCR primer-probe sets were selected based on literature review [9,10,12–15,50–52] and on the assays posted on WHO website [8] originally developed by seven different national institutions including Chinese Center for Disease Control and Prevention (China CDC), China; Institut Pasteur, Paris, France; US Centers for Disease Control and Prevention (CDC), USA; National Institute of Infectious Diseases, Japan; Charité – Universitätsmedizin Berlin Institute of Virology, Germany; The University of Hong Kong, Hong Kong; and National Institute of Health, Thailand.

### 2.2. Sequencing data

The complete genome sequences of the virus were downloaded from the Global Initiative on Sharing All Influenza Data (GISAID) EpiCoV database [53]. As of 7 May 2020, it hosted a total of 17 175 SARS-CoV-2 sequences isolated from humans. By applying the complete genome (greater than 29 000 bp) filter, a total of 17 026 sequences were included in the study that are available upon free registration (https://www.gisaid.org/). SARS-CoV-2 is an RNA virus, but the data are shown in DNA format as per scientific convention. The sequences are shared by the laboratories around the world and a list of accession numbers is included in electronic supplementary material, file S1. It is recognized that this study is not immune to the geographical bias present in academic and scientific research. As the data were sampled from a global sequence database, it is possible that data may originate from high-income countries like the literature in other disciplines [54,55]. In addition, it is possible that data from certain countries or regions are excluded based on the exclusion criteria of low-quality data that may skew the data geographically. Another reason for possible data skew may be the origin of the current pandemic being China. Indeed, a recent study analysed the publications in COVID-19 literature hub LitCovid [56] and observed that more than 30% of articles were related to China [57]. These aspects of possible bias and data skew are addressed in the Discussion to make sure that the valid conclusions are drawn from the data in terms of geographical correlation.

### 2.3. Multiple sequence alignment and alignment processing

Multiple sequence alignment (MSA) was performed using MAFFT (Multiple Alignment with Fast Fourier Transform) program v. 7 dedicated to closely related viral genomes [58,59] available online (https://mafft.cbrc.jp/alignment/server/). The complete genome of Wuhan-Hu-1 downloaded from NCBI on 7 May 2020 was included as a reference, which is 29 903 bp long (NCBI Reference Sequence: NC_045512.2). The aligned sequences were downloaded in PIR format. Each primer/probe was aligned with the MSA and the binding region referred to here as region of interest (ROI) was inspected using the AliView program 1.26 [60]. To evaluate the sequence variability in target regions of previously published primers/probes, the ROI for each primer/probe set was saved as a separate file in FASTA format.

### 2.4. Sequence variation in primer/probe binding regions in SARS-CoV-2 genome

The MSA sequence for forward primer, probe and reverse primer were stratified using the SequenceTracer module (http://entropy.szu.cz:8080/EntropyCalcWeb/sequences) of the Alignment Explorer [61]. This tool segregated sequences into discrete groups of identical sequence variants along with their frequency for each primer/probe. The sequences with stretches of NNNs, ambiguous sequences in ROI and missing sequences^1^ were excluded from the study. Subsequently, a threshold^2^ (0.5% of all sequences included) was defined to remove extremely low prevalent variants and sequencing errors in the data as described previously [61]. Thus, only the sequence variants with at least 0.5% incidence were further considered. The viral isolates were reported as the frequency of hits with perfect primer match and hits with mismatches along with a summary of mutated nucleotides for each primer/probe. The distribution of the sequence variants in three primers/probes with the highest frequency of mismatches were analysed geographically. As the sequence variation was moderate, the base composition of each nucleotide position was not analysed. As noted in the registered Stage 1 protocol (https://osf.io/ym8gc), this analysis can be performed using the positional nucleotide numerical summary (PNNS) calculator (http://entropy.szu.cz:8080/EntropyCalcWeb/pnns) of the Alignment Explorer [61].

## 3. Results

**Figure 2.**
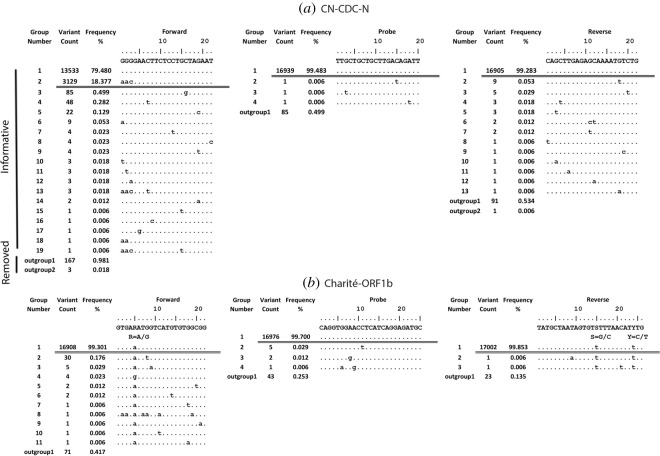
Sequence variants in primers and probe binding regions for CN-CDC-N (*a*) and Charité-ORF1b (*b*): sequence variants in 17 026 viral genome sequences aligned to the primer/probe binding regions (5′ → 3′) along with the number of sequence variants and the frequency of each variant in descending order. The dots indicate an identical nucleotide. The horizontal double bar indicates the threshold (greater than or equal to 0.5%). The binding region of reverse primer is reverse complemented. As an example, the removed and informative sequences are indicated with vertical bars. outgroup1, ambiguous sequences; outgroup2, short sequences.

**Figure 3.**
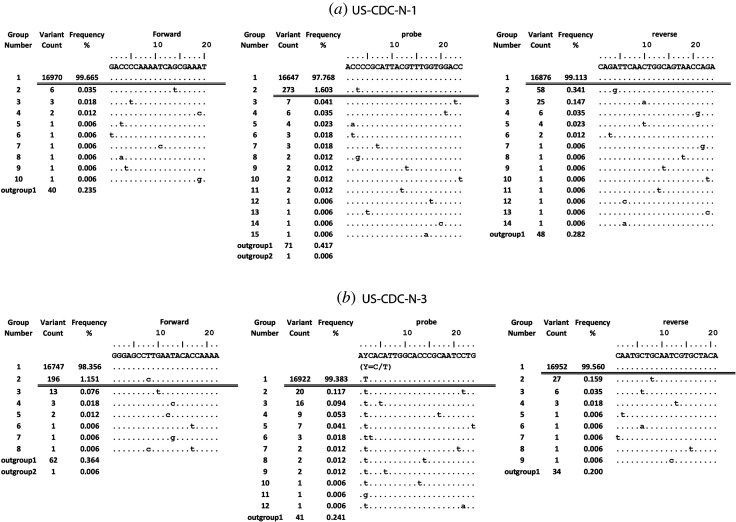
Sequence variants in primers and probe binding regions for US-CDC-N-1 (*a*) and US-CDC-N-3 (*b*): sequence variants in 17 026 viral genome sequences aligned to the primer/probe binding regions (5′→ 3′) along with the number of sequence variants and the frequency of each variant in descending order. The dots indicate an identical nucleotide. The horizontal double bar indicates the threshold (greater than or equal to 0.5%). The binding region of reverse primer is reverse complemented. outgroup1, ambiguous sequences; outgroup2, short sequences; excluded.

The sequence tracing pipeline (figure 1) was applied to the comprehensive sequence dataset of 17 027 SARS-CoV-2 sequences for each PCR primer/probe. To determine the sequence variability in the primer/probe binding regions, all the sequences in the dataset were aligned using MAFFT. Next, for each PCR assay, the MSA file was trimmed to include only the primer or probe binding regions referred to here as ROI. The sequence file for each primer/probe was submitted to SequenceTracer to segregate into discrete groups of identical sequence variants and presented a detailed view of the nucleotide variation in each ROI along with the frequency of each variant (figures 2 and 3; electronic supplementary material, file S2). All the sequences showing ambiguous sequences were grouped as ‘outgroup1’, short sequences were grouped as ‘outgroup2’ and missing sequences were grouped as ‘excluded’. These three groups were not included in the analysis (collectively referred here as ‘removed’), and the number of ‘informative’ sequences was calculated by subtracting these three groups from the total number of sequences. The informative group was then divided into hits with a perfect match and hits with mismatches for each primer and probe (table 2). It is not surprising that most primer/probe binding regions show mutations/mismatches with at least a couple of sequences but some of those may be extremely low prevalent variants and sequencing errors in the data. To minimize the effect of such sequences on the analysis, a threshold of 0.5% was then defined where only the sequence variants with at least 0.5% incidence were further considered as described previously [61]. The frequency of the sequences with the perfect match and with mismatches was then calculated from sequences above the threshold for each primer and probe. The summary of the analysis for 27 assays is presented in table 2.

**Table 2.** Reassesment of 27 published PCR diagnostic assays using 17 027 SARS-CoV-2 genome sequences.

gene target	assay name^a^	country	F/P/R^b^	sequence (5'–3′)	position^c^	total number of sequences				above threshold (≥0.5%)^d^			reference(s)
						removed	informative	perfect match	with mismatches	total	perfect match (%)	with mismatches (%)	
ORF1ab	Yip-ORF1ab	China	F	ATGCATTTGCATCAGAGGCT	1866–>1885	85	16 942	16 911	31	16 911	100		[14]
			R	TTGTTATAGCGGCCTTCTGT	1970<–1951	168	16 859	16 855	4	16 855	100		
	Pasteur-ORF1ab-1	France	F	ATGAGCTTAGTCCTGTTG	12 690–>12 707	54	16 973	16 973	0	16 973	100		[8]
			P	AGATGTCTTGTGCTGCCGGTA	12 717–>12 737	25	17 002	16 997	5	16 997	100		
			R	CTCCCTTTGTTGTGTTGT	12 797<–12 780	28	16 999	16 945	54	16 945	100		
	Pasteur-ORF1ab-2	France	F	GGTAACTGGTATGATTTCG	14 080–>14 098	46	16 981	16 981	0	16 981	100		[8]
			P	TCATACAAACCACGCCAGG	14 105–>14 123	45	16 982	16 958	24	16 958	100		
			R	CTGGTCAAGGTTAATATAGG	14 186<–14 167	50	16 977	16 939	38	16 939	100		
	CN-CDC-ORF1ab	China	F	CCCTGTGGGTTTTACACTTAA	13 342–>13 362	40	16 987	16 977	10	16 977	100		[8,12]
			P	CCGTCTGCGGTATGTGGAAAGGTTATGG	13 377–>13 404	1059	15 968	15 905	63	15 905	100		
			R	ACGATTGTGCATCAGCTGA	13 460<–13 442	1037	15 990	15 978	12	15 978	100		
	Young-ORF1ab	Singapore	F	TCATTGTTAATGCCTATATTAACC	14 155–>14 178	51	16 976	16 969	7	16 969	100		[15]
			P	AACTGCAGAGTCACATGTTGACA	14 193–>14 215	67	16 960	16 939	21	16 939	100		
			R	CACTTAATGTAAGGCTTTGTTAAG	14 243<–14 220	25	17 002	16 983	19	16 983	100		
	Charité-ORF1b	Germany	F	GTGARATGGTCATGTGTGGCGG	15 431–>15 452	71	16 956	16 908	48	16 908	100		[8,50]
			P	CAGGTGGAACCTCATCAGGAGATGC	15 470–>15 494	43	16 984	16 976	8	16 976	100		
			R	CARATGTTAAASACACTATTAGCATA	15 530<–15 505	23	17 004	0	17 004	17 002	0.0	100	
	Won-ORF1ab	South Korea	F	CATGTGTGGCGGTTCACTAT	15 441–>15 460	45	16 982	16 972	10	16 972	100		[13]
			R	TGCATTAACATTGGCCGTGA	15 558<-15 539	29	16 998	16 931	67	16 931	100		
	Chan-ORF1ab	China	F	CGCATACAGTCTTRCAGGCT	16 220–>16 239	69	16 958	16 946	12	16 946	100		[9]
			P	TTAAGATGTGGTGCTTGCATACGTAGAC	16 276–>16 303	84	16 943	16 786	157	16 930	99.1	0.9	
			R	GTGTGATGTTGAWATGACATGGTC	16 353<–16 330	86	16 941	0	16 941	16 932	0.0	100	
	HKU-ORF1b	Hong Kong	F	TGGGGYTTTACRGGTAACCT	18 778–>18 797	61	16 966	16 932	34	16 932	100		[8,52]
			P	TAGTTGTGATGCWATCATGACTAG	18 849–>18 872	41	16 986	16 976	10	16 976	100		
			R	AACRCGCTTAACAAAGCACTC	18 909<–18 889	48	16 979	16 958	21	16 958	100		
S	Young-S	Singapore	F	TATACATGTCTCTGGGACCA	21 763–>21 782	91	16 936	16 907	29	16 907	100		[15]
			P	CTAAGAGGTTTGATAACCCTGTCCTACC	21 789–>21 816	90	16 937	16 910	27	16 910	100		
			R	ATCCAGCCTCTTATTATGTTAGAC	21 876<–21 853	99	16 928	16 907	21	16 907	100		
	Chan-S	China	F	CCTACTAAATTAAATGATCTCTGCTTTACT	22 712–>22 741	254	16 773	16 768	5	16 768	100		[9]
			P	CGCTCCAGGGCAAACTGGAAAG	22 792–>22 813	262	16 765	16 752	13	16 752	100		
			R	CAAGCTATAACGCAGCCTGTA	22 869<–-22 849	65	16 962	16 956	6	16 956	100		
	Won-S	South Korea	F	CTACATGCACCAGCAACTGT	23 114->23 133	872	16 155	16 126	29	16 126	100		[13]
			R	CACCTGTGCCTGTTAAACCA	23 213<–23 194	29	16 998	16 987	11	16 987	100		
E	Won-E	South Korea	F	TTCGGAAGAGACAGGTACGTT	26 259–>26 279	33	16 994	16 986	8	16 986	100		[13]
			R	CACACAATCGATGCGCAGTA	26 365<–26 346	83	16 944	16 938	6	16 938	100		
	Charité-E	Germany	F	ACAGGTACGTTAATAGTTAATAGCGT	26 269–>26 294	47	16 980	16 975	5	16 975	100		[8,50]
			P	ACACTAGCCATCCTTACTGCGCTTCG	26 332–>26 357	75	16 952	16 928	24	16 928	100		
			R	ATATTGCAGCAGTACGCACACA	26 381<–26 360	89	16 938	16 928	10	16 928	100		
	Huang-E	China	F	ACTTCTTTTTCTTGCTTTCGTGGT	26 295–>26 318	80	16 947	16 925	22	16 925	100		[10]
			P	CTAGTTACACTAGCCATCCTTACTGC	26 326–>26 351	81	16 946	16 920	26	16 920	100		
			R	GCAGCAGTACGCACACAATC	26 376<–26 357	90	16 937	16 928	9	16 928	100		
	Niu-E	China	F	TTCTTGCTTTCGTGGTATTC	26 303–>26 322	78	16 949	16 926	23	16 926	100		[12]
			P	GTTACACTAGCCATCCTTACTGCGCTTCGA	26 329–>26 358	82	16 945	16 921	24	16 921	100		
			R	CACGTTAACAATATTGCAGC	26 391<–26 372	111	16 916	16 911	5	16 911	100		
N	CN-CDC-N	China	F	GGGGAACTTCTCCTGCTAGAAT	28 881–>28 902	170	16 857	13 533	3324	16 662	81.2	18.8	[8,12]
			P	TTGCTGCTGCTTGACAGATT	28 934–>28 953	85	16 942	16 939	3	16 939	100		
			R	CAGACATTTTGCTCTCAAGCTG	28 979<–28 958	92	16 935	16 905	30	16 905	100		
	NIH-TH_N	Thailand	F	CGTTTGGTGGACCCTCAGAT	28 320–>28 339	52	16 975	16 893	82	16 893	100		[8]
			P	CAACTGGCAGTAACCA	28 341–>28 356	42	16 985	16 946	39	16 946	100		
			R	CCCCACTGCGTTCTCCATT	28 376<–28 358	52	16 975	16 938	37	16 938	100		
	US-CDC-N-1	US	F	GACCCCAAAATCAGCGAAAT	28 287–>28 306	40	16 987	16 970	17	16 970	100		[8,62]
			P	ACCCCGCATTACGTTTGGTGGACC	28 309–>28 332	72	16 955	16 647	308	16 920	98.4	1.6	
			R	TCTGGTTACTGCCAGTTGAATCTG	28 358<–28 335	48	16 979	16 876	103	16 876	100		
	US-CDC-N-2	US	F	TTACAAACATTGGCCGCAAA	29 164–>29 183	339	16 688	16 647	41	16 647	100		[8,62]
			P	ACAATTTGCCCCCAGCGCTTCAG	29 188–>29 210	351	16 676	16 605	71	16 605	100		
			R	GCGCGACATTCCGAAGAA	29 230<–29 213	334	16 693	16 677	16	16 677	100		
	US-CDC-N-3	US	F	GGGAGCCTTGAATACACCAAAA	28 681–>28 702	63	16 964	16 747	217	16 943	98.8	1.2	[8,62]
			P	AYCACATTGGCACCCGCAATCCTG	28 704–>28 727	41	16 986	16 922	64	16 922	100		
			R	TGTAGCACGATTGCAGCATTG	28 752<–28 732	34	16 993	16 952	41	16 952	100		
	Young-N	Singapore	F	CTCAGTCCAAGATGGTATTTCT	28 583–>28 604	67	16 960	16 953	7	16 953	100		[15]
			P	ACCTAGGAACTGGCCCAGAAGCT	28 608–>28 630	58	16 969	0	16 969	16 927	0.0	100	
			R	AGCACCATAGGGAAGTCC	28 648<–28 631	52	16 975	16 949	26	16 949	100		
	Corman-N	Germany	F	CACATTGGCACCCGCAATC	28 706–>28 724	38	16 989	16 954	35	16 954	100		[50]
			P	ACTTCCTCAAGGAACAACATTGCCA	28 754–>28 777	75	16 952	16 930	22	16 930	100		
			R	GAGGAACGAGAAGAGGCTTG	28 833<–28 814	92	16 935	16 863	72	16 863	100		
	Won-N	South Korea	F	CAATGCTGCAATCGTGCTAC	28 732–>28 751	33	16 994	16 953	41	16 953	100		[13]
			R	GTTGCGACTACGTGATGAGG	28 849<–28 830	85	16 942	16 788	154	16 788	100		
	NIID-JP-N	Japan Japan	F	AAATTTTGGGGACCAGGAAC	29 125–>29 144	301	16 726	16 658	68	16 658	100		[8,51]
			P	ATGTCGCGCATTGGCATGGA	29 222–>29 241	329	16 698	16 679	19	16 679	100		
			R	TGGCAGCTGTGTAGGTCAAC	29 282<–29 263	309	16 718	0	16 718	16 687	0.0	100	
			R-v3	TGGCACCTGTGTAGGTCAAC	29 282<–29 263	309	16 718	16 687	31	16 687	100		[51]
	HKU-N	Hong Kong	F	TAATCAGACAAGGAACTGATTA	29 145–>29 166	309	16 718	16 667	51	16 667	100		[8,52]
			P	GCAAATTGTGCAATTTGCGG	29 177<–29 196	347	16 680	16 637	43	16 637	100		
			R	CGAAGGTGTGACTTCCATG	29 254<–29 236	320	16 707	16 668	39	16 668	100		
	Chan-N	China	F	GCGTTCTTCGGAATGTCG	29 210–>29 227	338	16 689	16 665	24	16 665	100		[9]
			P	AACGTGGTTGACCTACACAGST	29 257–>29 278	311	16 716	16 680	36	16 680	100		
			R	TTGGATCTTTGTCATCCAATTTG	29 306<–29 284	304	16 723	16 674	49	16 674	100		

^a^The assays were named in the following format: organization/author-gene target.

^b^ Forward primer (F), probe (P) and reverse primer (R).

^c^ Positions shown are with reference to NC_045 512.2.

^d^A threshold of 0.5% was defined where only the sequence variants with greater than or equal to 0.5% incidence were further considered.

It was observed that the primers/probe of 20 assays out of 27 assays tested showed a perfect match with the template at the defined threshold (table 2). It was further observed that the forward primer of CN-CDC-N showed three nucleotide mismatches with 18.8% of viral sequences (table 3 and figure 2*a*). In addition, the US-CDC-N-1 probe and the US-CDC-N-3 forward primer showed one mismatch with 1.6% and 1.2% viral sequences, respectively (table 3 and figure 3). The reverse primer of NIID-JP-N also showed one mismatch with all the sequences (table 3; electronic supplementary material, file S2). The probe of Chan-ORF1ab showed one mismatch with 0.9% of sequences while one mismatch in the reverse primer for all the sequences (table 3; electronic supplementary material, file S2). One mismatch was also observed with all the sequences for the probe of Young-N (table 3; electronic supplementary material, file S2). Most of the mismatches observed were not near the 3′ end of primers but some were in the probe binding regions. Many diagnostic assays have included degenerate nucleotides to increase the inclusivity of the assay for SARS-CoV and bat-SARS-related CoVs, but in certain cases, this is even detrimental for inclusive detection of SARS-CoV-2. For example, the Charité-ORF1b reverse primer contains an S (G or C) but all the viral sequences (in total 17 002) contain a T at this position (table 3 and figure 2*b*). Some of the other mutations observed in the primer/probe binding regions that did not pass the defined threshold include T13402G, C15540T, A28338G, C28846T, C28887T, C28896G, C29144T, T29148C and A29188T. Some of these are near the 3′ end of primers (figures 2 and 3; electronic supplementary material, file S2).

**Table 3.** Summary of primer/probe mismatches with SARS-CoV-2 genome.

primer name	F/P/R^b^	sequence (5′–3′)^c^ and suggested adjustment	genome position^d^	nucleotide		frequency
				primer	genome	
Charité-ORF1b	R	CARATGTTAAA**S**ACACTATTAGCATA Suggested modification from S to A (or R). CARATGTTAAA**A**ACACTATTAGCATA	15 519	S (G/C)^1^	T	17 002/17 002 (100%)
Chan-ORF1ab	P	TTAAGATGTGGTG**C**TTGCATACGTAGAC	16 289	C	T	144/16 930 (0.9%)
	R	**G**TGTGATGTTGAWATGACATGGTC Suggested modification from G to A **A**TGTGATGTTGAWATGACATGGTC	16 353	C^a^	T	16 932/16 932 (100%)
CN-CDC-N	F	**GGG**GAACTTCTCCTGCTAGAAT	28 881 28 882 28 883	GGG	AAC	3129/16 662 (18.8%)
US-CDC-N-1	P	AC**C**CCGCATTACGTTTGGTGGACC	29 311	C	T	273/16 920 (1.6%)
US-CDC-N-3	F	GGGAGCC**T**TGAATACACCAAAA	28 688	T	C	196/16 943 (1.2%)
Young-N	P	ACCTAGGAACTGG**C**CCAGAAGCT Suggested modification from C to G ACCTAGGAACTGG**G**CCAGAAGCT	28 621	C	G	16 969/16 969 (100%)
NIID-JP-N	R	TGGCA**G**CTGTGTAGGTCAAC Suggested modification from G to C [51] TGGCA**C**CTGTGTAGGTCAAC	29 277	C^a^	G	16 687/16 687 (100%)

^a^Reverse-complemented.

^b^Forward primer (F), probe (P) and reverse primer (R).

^c^Underlined and bold sequences indicate the mismatch observed and the suggested adjustment.

^d^Positions shown are with reference to NC_045512.2.

**Figure 4.**
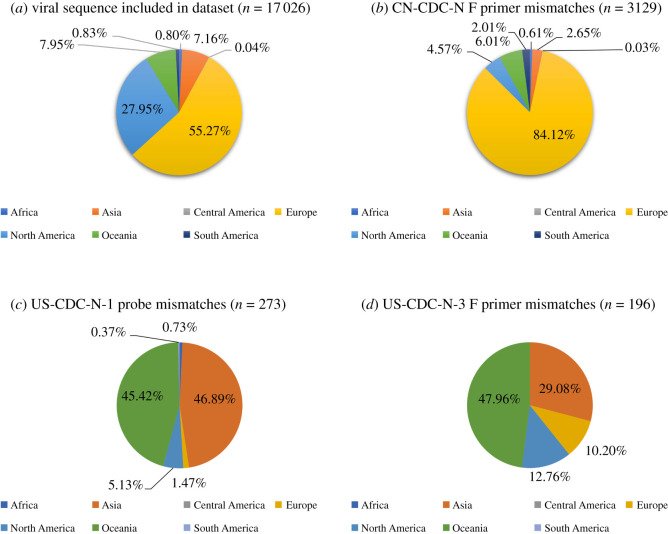
Geographical distribution of included sequences dataset (*a*) and mismatches for CN-CDC-N forward primer (*b*), US-CDC-N-1 probe (*c*) and US-CDC-N-3 forward primer (*d*). The total number of sequences in each dataset is given in parentheses. Data used to draw graphs are included in electronic supplementary material, file S3.

The majority of the sequences included in this study originated from Europe (9410) and North America (4759), while there were only 136 sequences from Africa, 7 from Central America and 142 from South America. The UK and the USA were among the countries with the highest number of sequences included (figure 4*a*; electronic supplementary material, file S3). The geographical distribution of the CN-CDC-N forward primer, US-CDC-N-1 probe and US-CDC-N-3 forward primer mismatches showed that it is distributed globally. However, mismatches with the CN-CDC-N forward primer were mostly found in Europe, while mismatches with the US-CDC-N-1 probe and the US-CDC-N-3 forward primer were found mostly in Australia and Asia (figure 4; electronic supplementary material, file S3).

## 4. Discussion

This study exhaustively evaluated the genetic diversity in the primer/probe binding regions of 27 previously published SARS-CoV-2 diagnostic assays including those recommended by WHO. The data presented in this study show mismatches in seven assays, highlighting the need for keeping the assay current through regular verification of sequence variation in PCR primer/probe binding regions. The other 20 assays show a perfect match with 100% of sequences at the defined threshold of 0.5%. This observation is in line with the estimates of the moderate mutation rate in the SARS-CoV-2 genome similar to the SARS-CoV genome [63,64]. It has been estimated that the mutation rate in the genome of coronaviruses is less than other RNA viruses while much higher than DNA viruses and the host [65,66]. Although all the sequences with mismatches were grouped in comparison to sequences with a perfect match, not all mismatches necessarily result in false-negative results. The effects of mismatch between primers/probes and template depend upon position and number of mismatches. Most of the mismatches observed in primers of SARS-CoV-2 diagnostic assays were not near the 3′ end and may be tolerated. Mismatches at the 3′ end are known for their deleterious effect on PCR amplification [17,67,68], but single mismatches, especially more than 5 bp far from the 3′ end, have a moderate effect on PCR amplification and are unlikely to significantly affect the assay performance [67]. Three assays showed a single nucleotide mismatch in the probe binding region. PCR amplification is more prone to mismatches in the probe region and even a single mismatch may reduce the sensitivity of the assay and lead to false-negative results due to the prevention of probe binding and subsequence fluorescence [22,28,69–71]. In the scenarios where mismatches were tolerated, one additional mutation resulted in reduced RT-qPCR sensitivity for the detection of influenza A virus [18].

Despite the ability of single mismatches to be tolerated, it is important to consider that mismatches need to be corrected if found in most of the viral sequences available. For example, the reverse primer of Charité-ORF1b shows a mismatch with all the viral sequences (a total of 17 002). This mismatch has also been observed in 990 viral sequences along with the lower sensitivity of this assay in a recent preprint [72]. Similarly, the NIID-JP-N reverse primer also shows a mismatch with all the sequences. This assay released by WHO was subsequently corrected by the authors in a separate study [51]. Although they show no difference in the performance of both assays, there is no apparent reason for not correcting the mismatch in the primer. The WHO recommended assays of SARS-CoV-2 were developed by multiple national organizations early in the outbreak with limited genomic sequence data available and have been instrumental for the diagnosis of COVID-19. However, some of the assays have not been reassessed in the light of the risk of mutations during viral evolution. Based on the analysis of 17 027 viral sequences, this study demonstrates the presence of mutations/mismatches in the primer/probe binding regions of some published assays (table 3). Sequences adjustments to these primers/probes need to be assessed experimentally using viral strains or nucleic acid coupled with subsequent experimental performance using clinical samples. With increasing concern of false-negative COVID-19 diagnosis and poor sensitivity of diagnostic PCR in certain cases [73,74], correcting the mismatches between primers/probes and template may help to improve the sensitivity of certain diagnostic assays.

There have been recent efforts along the same line where a limited number of viral sequences were aligned with primers/probes to search for mismatches. One of the recent preprints used 992 sequences to report some variants in the primer/probe binding regions [72]. However, many of the mismatches could be rare variants or sequencing errors, and variability in the assay binding regions should be assessed across a larger number of viral sequences. In addition, the diagnostic assay should not be revised based on the presence of rare variants in the population and thus a threshold of 0.5% was defined to eliminate such variants from the analysis. Some of the mismatches observed by this preprint were confirmed in the larger dataset of the current study. Other variants were not observed or did not reach the threshold and thus were not reported in the final analysis. It cannot be excluded that empirical threshold adjustment of this study might have missed some significant variants. For instance, choosing a threshold of 0.2% would have resulted in a mismatch with five additional assays that were reported to match with 100% of sequences in the current analysis. Another recent preprint reported a bioinformatics system named ‘BioLaboro’ to assess the efficacy of the existing PCR assays to detect pathogens as they evolve [75]. However, this system requires specialized software and large RAM hardware which is not generally available in regular diagnostic or research laboratories. By contrast, the current study validates a pipeline for *in silico* re-evaluation of PCR diagnostic assays of SARS-CoV-2. This approach has successfully been applied previously for influenza A virus [61]. Using freely available open-source software, the analysis was performed on a regular desktop computer without any need for special hardware. The pipeline does not require extensive computational skills except for some sequence alignment skills. The pipeline can be applied to a SARS-CoV-2 diagnostic assay of choice.

Verification of *in silico* nucleotide identity match, termed as inclusivity analysis, is also a component of the performance criteria of COVID-19 diagnostic assays by the U.S. Food and Drug Administration (FDA) as well as the European Commission [76,77]. Several commercially developed COVID-19 diagnostic assays have received limited regulatory approval due to the emergency situation. As of 12 May 2020, a total of 54 commercial diagnostic test kits including the one developed by the US-CDC have received emergency use authorization (EUA) from the FDA [78]. The CDC has also reported one nucleotide mismatch in the N1 forward primer in their inclusivity assay using sequences available as of 1 February 2020 [62]. Some commercial kits like BD BioGX use CDC primers and thus do not conduct independent inclusivity analysis [79]. Many other kits have reported the alignment of their assay primers/probes with a couple of hundred sequences [80–85]. As primer/probe identity for most commercial kits is not revealed, manufacturer-independent data are scarce. Recent comparisons of SARS-CoV-2 diagnostic assays have shown some discordance which may partially be due to sequence differences [86,87]. Therefore, there is a need for comprehensive inclusivity assessment of commercial diagnostic assays. Although not addressed in this article, other factors for reassessment include *in silico* cross-reactivity with human genes, genes of other members of family Coronaviridae and other respiratory viruses/bacteria.

The methodology outlined here uses MSA of publicly available viral sequences and is prone to certain biases despite its general utility in diagnostic PCR assay design. One of the biases is the compositional bias, which may arise as a result of sampling from certain geographical locations due to access to better facilities for viral genome sequencing or location of the outbreak. Based on a relatively moderate mutation rate in the genome, the results obtained can be applied globally, but caution should be exercised when drawing conclusions from the results for a specific region, especially with a smaller number of sequences included. Another possible geographical bias can arise due to the removal of data collected from certain countries or regions. However, the fact that less than 2.1% of sequences were removed for 73 out of 76 primers/probes studied mitigates this concern in the current study. The geographical analysis of the removed data (approx. 6%) of the remaining three primers/probes showed that most of the removed viral sequences were from Europe as expected (electronic supplementary material, file S4). Although the risk of data skew geographically cannot be ruled out completely, this much data exclusion is in line with previous reports [61]. Another source of compositional bias may be the redundancy where the same viral strain is re-sequenced and re-submitted to the sequence database.

Another source of bias may arise from the submission of isolates after passaging in the cell culture as well as sequencing artefacts including ambiguous data, short artificial insertions or deletions, incorrect sequence directions, incorrect nucleotide insertions, short sequence stretches and sequence longer than standard length [88]. Most data in the EpiCov database include the full-length data, and thus short sequences were not included in the study. To remove artificially inserted sequences and sequences at the ends, if any, MSA was performed with the option to keep the alignment length according to the reference sequence. In this methodology, no gaps are inserted in the reference sequence and corresponding sites in the other sequences are deleted. Therefore, this methodology can potentially remove any real insertions as well. However, only seven insertions affecting 31 sequences are catalogued in CoV-GLUE database (http://cov-glue.cvr.gla.ac.uk/#/insertion) as of 22 May 2020 [89]. The use of SequenceTracer in the tracing pipeline successfully filters out ambiguous data and deletions [61]. As SequenceTracer removes all the sequences with short and missing sequences, a real deletion of a stretch of sequence would also be filtered out. However, only a few sequences were removed in the ‘outgroup2’ or in ‘excluded’ group (figures 2 and 3; electronic supplementary material, file S2). In line, none of the deletions affecting more than two sequences listed in CoV-GLUE database (http://cov-glue.cvr.gla.ac.uk/#/deletion) as of 22 May 2020 were found in the ROI under study.

## 5. Conclusion

This work outlines a comprehensive approach for the bioinformatics reassessment of PCR diagnostic assays for SARS-CoV-2. The application of this strategy on 27 previously developed assays using 17 027 viral sequences showed mutations/mismatches in primer/probe binding regions of seven assays. This information will act as a reference and may help re-evaluate COVID-19 diagnostic strategies. *In silico* analysis of primers/probes should be coupled with empirical testing on clinical samples and the primers/probes that work well *in silico* as well as empirically should be used in a diagnostic assay for SARS-CoV-2.

## Supplementary Material

Acknowledgement table

Reviewer comments

## Supplementary Material

Sequence tracing figures

## Supplementary Material

Geographical distribution of included sequence dataset and mismatches

## Supplementary Material

Geographical distribution of removed data

## Footnotes

1 SequenceTracer removes the missing sequences in ROI. The exclusion criterion of missing sequences was clarified with editorial approval after Stage 1 acceptance and prior to observation of the data.

2 The threshold was decided before Stage 1 acceptance. However, it was not clearly mentioned in the Stage 1 protocol and a previous study was referenced only.

## Data accessibility

A list of accession numbers of sequences is included in electronic supplementary material, file S1. Sequence tracing figures of all the assays not shown in the main article are included in electronic supplementary material, file S2. Geographical data used to draw graphs in figure 4 are included in electronic supplementary material, file S3. The geographical analysis of removed data for three primers/probe with the highest frequency is included in electronic supplementary material, file S4.
