# Supplementary material for: Presence of mismatches between diagnostic PCR assays and coronavirus SARS-CoV-2 genome
Source: R Soc Open Sci. 2020 Jun 10;7(6):200636. doi: 10.1098/rsos.200636 (PMC7353963; doi:10.1098/rsos.200636)
Supplement: Sequence tracing figures [file rsos200636supp2.pdf]

Cite this article: Khan KA, Cheung P. 2020 Presence of mismatches between diagnostic PCR assays and coronavirus SARS-CoV-2 genome. R. Soc. Open Sci. 7: 200636. http://dx.doi.org/10.1098/rsos.200636

Sequence tracing figures for 23 previously published SARS-CoV-2 diagnostic assay (not shown in the main article): Sequence variants in 17026 viral genome sequences aligned to the primers/probe binding regions (5′ → 3′) along with the number of sequence variants and the frequency of each variant in a descending order. The complete genome of Wuhan-Hu-1 downloaded from NCBI was included as a reference (NCBI Reference Sequence: NC_045512.2). The binding region of reverse primer is reverse complemented. The dots indicate an identical nucleotide. The horizontal double bar indicates the threshold (≥0.5%). outgroup1, ambiguous sequences; outgroup2, short sequences; excluded, missing sequences.

Yip-ORF1ab

<table>
<tr><th>Group Number</th><th>Variant Count</th><th>Frequency %</th><th>Forward
10 20
....|....|....|....|
ATGCATTTGCATCAGAGGCT</th></tr>
<tr><td>1</td><td>16911</td><td>99.319</td><td>....................</td></tr>
<tr><td>2</td><td>16</td><td>0.094</td><td>..................t.</td></tr>
<tr><td>3</td><td>7</td><td>0.041</td><td>................t...</td></tr>
<tr><td>4</td><td>3</td><td>0.018</td><td>............t.......</td></tr>
<tr><td>5</td><td>3</td><td>0.018</td><td>...........c........</td></tr>
<tr><td>6</td><td>1</td><td>0.006</td><td>........a...........</td></tr>
<tr><td>7</td><td>1</td><td>0.006</td><td>g...................</td></tr>
<tr><td>outgroup1</td><td>85</td><td>0.499</td><td></td></tr>
</table>

<table>
<tr><th>Group Number</th><th>Variant Count</th><th>Frequency %</th><th>Reverse
10 20
....|....|....|....|
ACAGAAGGCCGCTATAACAA</th></tr>
<tr><td>1</td><td>16855</td><td>98.990</td><td>....................</td></tr>
<tr><td>2</td><td>2</td><td>0.012</td><td>.................t..</td></tr>
<tr><td>3</td><td>1</td><td>0.006</td><td>......t.............</td></tr>
<tr><td>4</td><td>1</td><td>0.006</td><td>.........t..........</td></tr>
<tr><td>outgroup1</td><td>168</td><td>0.987</td><td></td></tr>
</table>

Pasteur-ORF1ab-1

Group Number	Variant Count	Frequency %	Forward
			10
			....|....|....|...
			ATGAGCTTAGTCCTGTTG
1	16973	99.683	....
outgroup1	54	0.317	

Group Number	Variant Count	Frequency %	Probe
			10 20
			....|....|....|....|.
			AGATGTCTTGTGCTGCCGGTA
1	16997	99.824	....
2	4	0.023	.....c...............
3	1	0.006	................t....
outgroup1	24	0.141	
outgroup2	1	0.006	

Group Number	Variant Count	Frequency %	Reverse
			10
			....|....|....|...
			ACAACACAACAAAGGGAG
1	16945	99.518	....
2	21	0.123	.t................
3	18	0.106	..........g.......
4	7	0.041	................c.
5	2	0.012	.................a
6	2	0.012	.............t....
7	1	0.006	......t...........
8	1	0.006	................g.
9	1	0.006	............t.t.t.
10	1	0.006	.......g..........
outgroup1	28	0.164	

Pasteur-ORF1ab-2

Group Number	Variant Count	Frequency %	Forward
			10
			....|....|....|....
			GGTAACTGGTATGATTTCG
1	16981	99.730	....
outgroup1	46	0.270	

Group Number	Variant Count	Frequency %	Probe
			10
			....|....|....|....
			TCATACAAACCACGCCAGG
1	16958	99.595	....
2	12	0.070	.............t.....
3	6	0.035	...............t...
4	3	0.018	.................t.
5	3	0.018	.............a.....
outgroup1	45	0.264	

Group Number	Variant Count	Frequency %	Reverse
			10 20
			....|....|....|....|
			CCTATATTAACCTTGACCAG
1	16939	99.483	....
2	16	0.094	.................t..
3	8	0.047	..................t.
4	6	0.035	................t...
5	4	0.023	...........t........
6	2	0.012	...................a
7	1	0.006	...t................
8	1	0.006	.a..................
outgroup1	50	0.294	

CN-CDC-ORF1ab

<table>
<tr><th>Group Number</th><th>Variant Count</th><th>Frequency %</th><th>Forward</th></tr>
<tr><td></td><td></td><td></td><td>10 20</td></tr>
<tr><td></td><td></td><td></td><td>....|....|....|....|.</td></tr>
<tr><td></td><td></td><td></td><td>CCCTGTGGGTTTTACACTTAA</td></tr>
<tr><td>1</td><td>16977</td><td>99.706</td><td>.....................</td></tr>
<tr><td>2</td><td>4</td><td>0.023</td><td>......t..............</td></tr>
<tr><td>3</td><td>2</td><td>0.012</td><td>...c.................</td></tr>
<tr><td>4</td><td>2</td><td>0.012</td><td>................t....</td></tr>
<tr><td>5</td><td>2</td><td>0.012</td><td>..............t......</td></tr>
<tr><td>outgroup1</td><td>40</td><td>0.235</td><td></td></tr>
</table>

<table>
<tr><th>Group Number</th><th>Variant Count</th><th>Frequency %</th><th>Probe</th></tr>
<tr><td></td><td></td><td></td><td>10 20</td></tr>
<tr><td></td><td></td><td></td><td>....|....|....|....|....|...</td></tr>
<tr><td></td><td></td><td></td><td>CCGTCTGCGGTATGTGGAAAGGTTATGG</td></tr>
<tr><td>1</td><td>15905</td><td>93.410</td><td>............................</td></tr>
<tr><td>2</td><td>52</td><td>0.305</td><td>.........................g..</td></tr>
<tr><td>3</td><td>2</td><td>0.012</td><td>.t..........................</td></tr>
<tr><td>4</td><td>2</td><td>0.012</td><td>........................t...</td></tr>
<tr><td>5</td><td>1</td><td>0.006</td><td>at..........................</td></tr>
<tr><td>6</td><td>1</td><td>0.006</td><td>......c.....................</td></tr>
<tr><td>7</td><td>1</td><td>0.006</td><td>.............t..t.c.........</td></tr>
<tr><td>8</td><td>1</td><td>0.006</td><td>................t...........</td></tr>
<tr><td>9</td><td>1</td><td>0.006</td><td>a...........................</td></tr>
<tr><td>10</td><td>1</td><td>0.006</td><td>.......t....................</td></tr>
<tr><td>11</td><td>1</td><td>0.006</td><td>....t.......................</td></tr>
<tr><td>outgroup1</td><td>1059</td><td>6.220</td><td></td></tr>
</table>

<table>
<tr><th>Group Number</th><th>Variant Count</th><th>Frequency %</th><th>Reverse</th></tr>
<tr><td></td><td></td><td></td><td>10</td></tr>
<tr><td></td><td></td><td></td><td>....|....|....|....</td></tr>
<tr><td></td><td></td><td></td><td>TCAGCTGATGCACAATCGT</td></tr>
<tr><td>1</td><td>15978</td><td>93.839</td><td>...................</td></tr>
<tr><td>2</td><td>5</td><td>0.029</td><td>................t..</td></tr>
<tr><td>3</td><td>1</td><td>0.006</td><td>....a..............</td></tr>
<tr><td>4</td><td>1</td><td>0.006</td><td>...........c..t....</td></tr>
<tr><td>5</td><td>1</td><td>0.006</td><td>.........t.........</td></tr>
<tr><td>6</td><td>1</td><td>0.006</td><td>........a..........</td></tr>
<tr><td>7</td><td>1</td><td>0.006</td><td>...c...............</td></tr>
<tr><td>8</td><td>1</td><td>0.006</td><td>...a...............</td></tr>
<tr><td>9</td><td>1</td><td>0.006</td><td>...t...............</td></tr>
<tr><td>outgroup1</td><td>1037</td><td>6.090</td><td></td></tr>
</table>

Young-ORF1ab

<table>
<tr><th>Group Number</th><th>Variant Count</th><th>Frequency %</th><th>Forward</th></tr>
<tr><td></td><td></td><td></td><td>10 20</td></tr>
<tr><td></td><td></td><td></td><td>....|....|....|....|....</td></tr>
<tr><td></td><td></td><td></td><td>TCATTGTTAATGCCTATATTAACC</td></tr>
<tr><td>1</td><td>16969</td><td>99.659</td><td>........................</td></tr>
<tr><td>2</td><td>4</td><td>0.023</td><td>.......................t</td></tr>
<tr><td>3</td><td>1</td><td>0.006</td><td>...............t........</td></tr>
<tr><td>4</td><td>1</td><td>0.006</td><td>...c....................</td></tr>
<tr><td>5</td><td>1</td><td>0.006</td><td>.............a..........</td></tr>
<tr><td>outgroup1</td><td>51</td><td>0.300</td><td></td></tr>
</table>

<table>
<tr><th>Group Number</th><th>Variant Count</th><th>Frequency %</th><th>Probe</th></tr>
<tr><td></td><td></td><td></td><td>10 20</td></tr>
<tr><td></td><td></td><td></td><td>....|....|....|....|...</td></tr>
<tr><td></td><td></td><td></td><td>AACTGCAGAGTCACATGTTGACA</td></tr>
<tr><td>1</td><td>16939</td><td>99.483</td><td>.......................</td></tr>
<tr><td>2</td><td>5</td><td>0.029</td><td>..a....................</td></tr>
<tr><td>3</td><td>4</td><td>0.023</td><td>.....t.................</td></tr>
<tr><td>4</td><td>3</td><td>0.018</td><td>......g................</td></tr>
<tr><td>5</td><td>2</td><td>0.012</td><td>.....................t.</td></tr>
<tr><td>6</td><td>1</td><td>0.006</td><td>................t......</td></tr>
<tr><td>7</td><td>1</td><td>0.006</td><td>................a......</td></tr>
<tr><td>8</td><td>1</td><td>0.006</td><td>...................a...</td></tr>
<tr><td>9</td><td>1</td><td>0.006</td><td>....a..................</td></tr>
<tr><td>10</td><td>1</td><td>0.006</td><td>....t..................</td></tr>
<tr><td>11</td><td>1</td><td>0.006</td><td>..................c....</td></tr>
<tr><td>12</td><td>1</td><td>0.006</td><td>...c...................</td></tr>
<tr><td>outgroup1</td><td>66</td><td>0.388</td><td></td></tr>
<tr><td>outgroup2</td><td>1</td><td>0.006</td><td></td></tr>
</table>

<table>
<tr><th>Group Number</th><th>Variant Count</th><th>Frequency %</th><th>Reverse</th></tr>
<tr><td></td><td></td><td></td><td>10 20</td></tr>
<tr><td></td><td></td><td></td><td>....|....|....|....|....</td></tr>
<tr><td></td><td></td><td></td><td>CTTAACAAAGCCTTACATTAAGTG</td></tr>
<tr><td>1</td><td>16983</td><td>99.742</td><td>........................</td></tr>
<tr><td>2</td><td>7</td><td>0.041</td><td>.....................t..</td></tr>
<tr><td>3</td><td>6</td><td>0.035</td><td>t.......................</td></tr>
<tr><td>4</td><td>2</td><td>0.012</td><td>...............t........</td></tr>
<tr><td>5</td><td>1</td><td>0.006</td><td>..gc.t..................</td></tr>
<tr><td>6</td><td>1</td><td>0.006</td><td>..........t.............</td></tr>
<tr><td>7</td><td>1</td><td>0.006</td><td>.........t..............</td></tr>
<tr><td>8</td><td>1</td><td>0.006</td><td>....g...................</td></tr>
<tr><td>outgroup1</td><td>25</td><td>0.147</td><td></td></tr>
</table>

Won-ORF1ab

Group Number	Variant Count	Frequency %	Forward
			10 20
			....|....|....|....|
			CATGTGTGGCGGTTCACTAT
1	16972	99.677	....
2	2	0.012	.........t..........
3	2	0.012	...t................
4	1	0.006	.......t............
5	1	0.006	a.......a...........
6	1	0.006	..........a.........
7	1	0.006	t...................
8	1	0.006	.......a............
9	1	0.006	c
outgroup1	45	0.264	

Group Number	Variant Count	Frequency %	Reverse
			10 20
			....|....|....|....|
			TCACGGCCAATGTTAATGCA
1	16931	99.436	....
2	61	0.358	.t..................
3	2	0.012	....t...............
4	1	0.006	c...................
5	1	0.006	....a...............
6	1	0.006	..............g.....
7	1	0.006	.......a............
outgroup1	29	0.170	

Chan-ORF1ab

Group Number	Variant Count	Frequency %	Forward
			10 20
			....|....|....|....|
			CGCATACAGTCTTRCAGGCT
1			R=A/G
2	16946	99.524	.............a......
3	4	0.023	.a...........a......
4	4	0.023	..t..........a......
5	2	0.012	.t...........a......
outgroup1	2	0.012	.............a..t...
	69	0.405	

Group Number	Variant Count	Frequency %	Probe
			10 20
			....|....|....|....|....|...
			TTAAGATGTGGTGCTTGCATACGTAGAC
1	16786	98.585	....
2	144	0.846	.............t..............
3	10	0.059	.................t..........
4	1	0.006	...................c........
5	1	0.006	.......................c....
6	1	0.006	...........................t
outgroup1	84	0.493	

Group Number	Variant Count	Frequency %	Reverse
			10 20
			....|....|....|....|....
			GACCATGTCATWTCAACATCACAC
			W=A/T
1	16932	99.442	...........a...........t
2	3	0.018	........t..a...........t
3	3	0.018	...........ac..........t
4	1	0.006	........a..a...........t
5	1	0.006	.....g.....a...........t
6	1	0.006	...........a.t.........t
outgroup1	86	0.505	

HKU-ORF1b

Forward

```
10        20
....|....|....|....|
TGGGGYTTTACRGGTAACCT
  Y=C/T R=A/G
```

Group Number	Variant Count	Frequency %	Forward
1	16932	99.442	.....t.....a........
2	28	0.164	.....t....ta........
3	2	0.012	....tt.....a........
4	1	0.006	...t.t.....a........
5	1	0.006	.....t.....a....t...
6	1	0.006	.....t.....c........
7	1	0.006	..t..t.....a........
outgroup1	61	0.358	

Probe

```
10        20
....|....|....|....|....
TAGTTGTGATGCWATCATGACTAG
W=A/T
```

Group Number	Variant Count	Frequency %	Probe
1	16976	99.700	............a...........
2	8	0.047	..........t.t...........
3	1	0.006	............t.c.........
4	1	0.006	...........at...........
outgroup1	41	0.241	

Reverse

```
10        20
....|....|....|....|.
GAGTGCTTTGTTAAGCGYGTT
Y=C/T
```

Group Number	Variant Count	Frequency %	Reverse
1	16958	99.595	.................t...
2	7	0.041	...............t.t...
3	6	0.035	.................tt..
4	4	0.023	.....t...........t...
5	3	0.018	.........t.......t...
6	1	0.006	.................t..c
outgroup1	48	0.282	

Young-S

Forward

```
10        20
....|....|....|....|
TATACATGTCTCTGGGACCA
```

Group Number	Variant Count	Frequency %	Forward
1	16907	99.295	....
2	7	0.041	.........t..........
3	4	0.023	...........t........
4	4	0.023	...............a....
5	3	0.018	............c.......
6	3	0.018	....t...............
7	2	0.012	..................t.
8	2	0.012	...............t....
9	1	0.006	.......t............
10	1	0.006	..................a.
11	1	0.006	....g....ta..t..tt.c
12	1	0.006	.............t......
outgroup1	90	0.529	
excluded	1	0.006	

Probe

```
10        20
....|....|....|....|....|...
CTAAGAGGTTTGATAACCCTGTCCTACC
```

Group Number	Variant Count	Frequency %	Probe
1	16910	99.313	....
2	8	0.047	...........t................
3	6	0.035	t...........................
4	3	0.018	...t........................
5	2	0.012	......................t.....
6	2	0.012	......a.....................
7	1	0.006	.............c..............
8	1	0.006	..............g.............
9	1	0.006	...g......atca..............
10	1	0.006	....t.......................
11	1	0.006	.......a....................
12	1	0.006	....................t.......
outgroup1	90	0.529	

Reverse

```
10        20
....|....|....|....|....
GTCTAACATAATAAGAGGCTGGAT
```

Group Number	Variant Count	Frequency %	Reverse
1	16907	99.295	....
2	9	0.053	..t.....................
3	4	0.023	......t.................
4	3	0.018	..............t.........
5	2	0.012	a.......................
6	1	0.006	...................g....
7	1	0.006	...............t........
8	1	0.006	...................a....
outgroup1	99	0.581	

Chan-S

<table>
<tr><th>Group Number</th><th>Variant Count</th><th>Frequency %</th><th>Forward
10 20 30</th></tr>
<tr><td></td><td></td><td></td><td>....|....|....|....|....|....|
CCTACTAAATTAAATGATCTCTGCTTTACT</td></tr>
<tr><td>1</td><td>16768</td><td>98.479</td><td>..............................</td></tr>
<tr><td>2</td><td>4</td><td>0.023</td><td>.t............................</td></tr>
<tr><td>3</td><td>1</td><td>0.006</td><td>.......................t......</td></tr>
<tr><td>outgroup1</td><td>254</td><td>1.492</td><td></td></tr>
</table>

<table>
<tr><th>Group Number</th><th>Variant Count</th><th>Frequency %</th><th>Probe
10 20</th></tr>
<tr><td></td><td></td><td></td><td>....|....|....|....|..
CGCTCCAGGGCAAACTGGAAAG</td></tr>
<tr><td>1</td><td>16752</td><td>98.385</td><td>......................</td></tr>
<tr><td>2</td><td>6</td><td>0.035</td><td>..........g...........</td></tr>
<tr><td>3</td><td>2</td><td>0.012</td><td>.........t............</td></tr>
<tr><td>4</td><td>1</td><td>0.006</td><td>t.....................</td></tr>
<tr><td>5</td><td>1</td><td>0.006</td><td>..........gc..........</td></tr>
<tr><td>6</td><td>1</td><td>0.006</td><td>..............t.......</td></tr>
<tr><td>7</td><td>1</td><td>0.006</td><td>......g...............</td></tr>
<tr><td>8</td><td>1</td><td>0.006</td><td>a.....................</td></tr>
<tr><td>outgroup1</td><td>261</td><td>1.533</td><td></td></tr>
<tr><td>outgroup2</td><td>1</td><td>0.006</td><td></td></tr>
</table>

<table>
<tr><th>Group Number</th><th>Variant Count</th><th>Frequency %</th><th>Reverse
10 20</th></tr>
<tr><td></td><td></td><td></td><td>....|....|....|....|.
TACAGGCTGCGTTATAGCTTG</td></tr>
<tr><td>1</td><td>16956</td><td>99.583</td><td>.....................</td></tr>
<tr><td>2</td><td>3</td><td>0.018</td><td>.........t...........</td></tr>
<tr><td>3</td><td>1</td><td>0.006</td><td>......a..a..a.a......</td></tr>
<tr><td>4</td><td>1</td><td>0.006</td><td>................t....</td></tr>
<tr><td>5</td><td>1</td><td>0.006</td><td>...............g.....</td></tr>
<tr><td>outgroup1</td><td>64</td><td>0.376</td><td></td></tr>
<tr><td>outgroup2</td><td>1</td><td>0.006</td><td></td></tr>
</table>

Won-S

<table>
<tr><th>Group Number</th><th>Variant Count</th><th>Frequency %</th><th>Forward
10 20</th></tr>
<tr><td></td><td></td><td></td><td>....|....|....|....|
CTACATGCACCAGCAACTGT</td></tr>
<tr><td>1</td><td>16126</td><td>94.708</td><td>....................</td></tr>
<tr><td>2</td><td>7</td><td>0.041</td><td>......t.............</td></tr>
<tr><td>3</td><td>4</td><td>0.023</td><td>t...................</td></tr>
<tr><td>4</td><td>4</td><td>0.023</td><td>..t.................</td></tr>
<tr><td>5</td><td>4</td><td>0.023</td><td>............t.......</td></tr>
<tr><td>6</td><td>4</td><td>0.023</td><td>.............t......</td></tr>
<tr><td>7</td><td>2</td><td>0.012</td><td>.....a..............</td></tr>
<tr><td>8</td><td>1</td><td>0.006</td><td>...........g........</td></tr>
<tr><td>9</td><td>1</td><td>0.006</td><td>........t...........</td></tr>
<tr><td>10</td><td>1</td><td>0.006</td><td>....c...............</td></tr>
<tr><td>11</td><td>1</td><td>0.006</td><td>.........t..........</td></tr>
<tr><td>outgroup1</td><td>871</td><td>5.115</td><td></td></tr>
<tr><td>outgroup2</td><td>1</td><td>0.006</td><td></td></tr>
</table>

<table>
<tr><th>Group Number</th><th>Variant Count</th><th>Frequency %</th><th>Reverse
10 20</th></tr>
<tr><td></td><td></td><td></td><td>....|....|....|....|
TGGTTTAACAGGCACAGGTG</td></tr>
<tr><td>1</td><td>16987</td><td>99.765</td><td>....................</td></tr>
<tr><td>2</td><td>5</td><td>0.029</td><td>........t...........</td></tr>
<tr><td>3</td><td>4</td><td>0.023</td><td>..............t.....</td></tr>
<tr><td>4</td><td>2</td><td>0.012</td><td>............t.......</td></tr>
<tr><td>outgroup1</td><td>29</td><td>0.170</td><td></td></tr>
</table>

Won-E

Group Number	Variant Count	Frequency %	Forward
			10 20
			....|....|....|....|.
			TTCGGAAGAGACAGGTACGTT
1	16986	99.759	....
2	2	0.012	...........t.........
3	2	0.012	...a.................
4	1	0.006	...............c.....
5	1	0.006	..t..................
6	1	0.006	....c................
7	1	0.006	.......a.............
outgroup1	33	0.194	

Group Number	Variant Count	Frequency %	Reverse
			10 20
			....|....|....|....|
			TACTGCGCTTCGATTGTGTG
1	16938	99.477	....
2	2	0.012	...................t
3	1	0.006	.....t..............
4	1	0.006	........g...........
5	1	0.006	........a...........
6	1	0.006	.......t............
outgroup1	79	0.464	
outgroup2	3	0.018	
excluded	1	0.006	

Charité-E

Group Number	Variant Count	Frequency %	Forward
			10 20
			....|....|....|....|....|.
			ACAGGTACGTTAATAGTTAATAGCGT
1	16975	99.695	....
2	2	0.012	.t........................
3	1	0.006	............g.............
4	1	0.006	c
5	1	0.006	.....c....................
outgroup1	46	0.270	
outgroup2	1	0.006	

Group Number	Variant Count	Frequency %	Probe
			10 20
			....|....|....|....|....|.
			ACACTAGCCATCCTTACTGCGCTTCG
1	16928	99.419	....
2	17	0.100	........t.................
3	3	0.018	.t........................
4	1	0.006	...................t......
5	1	0.006	......................g...
6	1	0.006	......................a...
7	1	0.006	.....................t....
outgroup1	73	0.429	
outgroup2	2	0.012	

Group Number	Variant Count	Frequency %	Reverse
			10 20
			....|....|....|....|..
			TGTGTGCGTACTGCTGCAATAT
1	16928	99.419	....
2	6	0.035	..........t...........
3	2	0.012	.....t................
4	1	0.006	.............g........
5	1	0.006	....................t.
outgroup1	86	0.505	
outgroup2	1	0.006	
excluded	2	0.012	

Huang-E

<table>
<tr><th>Group Number</th><th>Variant Count</th><th>Frequency %</th><th>Forward</th></tr>
<tr><td></td><td></td><td></td><td>10 20</td></tr>
<tr><td></td><td></td><td></td><td>....|....|....|....|....</td></tr>
<tr><td></td><td></td><td></td><td>ACTTCTTTTTCTTGCTTTCGTGGT</td></tr>
<tr><td>1</td><td>16925</td><td>99.401</td><td>........................</td></tr>
<tr><td>2</td><td>7</td><td>0.041</td><td>...................a....</td></tr>
<tr><td>3</td><td>5</td><td>0.029</td><td>......c.................</td></tr>
<tr><td>4</td><td>3</td><td>0.018</td><td>..................t.....</td></tr>
<tr><td>5</td><td>2</td><td>0.012</td><td>......g.................</td></tr>
<tr><td>6</td><td>1</td><td>0.006</td><td>.............t..........</td></tr>
<tr><td>7</td><td>1</td><td>0.006</td><td>..............t.........</td></tr>
<tr><td>8</td><td>1</td><td>0.006</td><td>..........t.............</td></tr>
<tr><td>9</td><td>1</td><td>0.006</td><td>.t......................</td></tr>
<tr><td>10</td><td>1</td><td>0.006</td><td>...................t....</td></tr>
<tr><td>outgroup1</td><td>79</td><td>0.464</td><td></td></tr>
<tr><td>outgroup2</td><td>1</td><td>0.006</td><td></td></tr>
</table>

<table>
<tr><th>Group Number</th><th>Variant Count</th><th>Frequency %</th><th>Probe</th></tr>
<tr><td></td><td></td><td></td><td>10 20</td></tr>
<tr><td></td><td></td><td></td><td>....|....|....|....|....|.</td></tr>
<tr><td></td><td></td><td></td><td>CTAGTTACACTAGCCATCCTTACTGC</td></tr>
<tr><td>1</td><td>16920</td><td>99.372</td><td>..........................</td></tr>
<tr><td>2</td><td>17</td><td>0.100</td><td>..............t...........</td></tr>
<tr><td>3</td><td>5</td><td>0.029</td><td>t.........................</td></tr>
<tr><td>4</td><td>3</td><td>0.018</td><td>.......t..................</td></tr>
<tr><td>5</td><td>1</td><td>0.006</td><td>.........................t</td></tr>
<tr><td>outgroup1</td><td>79</td><td>0.464</td><td></td></tr>
<tr><td>outgroup2</td><td>2</td><td>0.012</td><td></td></tr>
</table>

<table>
<tr><th>Group Number</th><th>Variant Count</th><th>Frequency %</th><th>Reverse</th></tr>
<tr><td></td><td></td><td></td><td>10 20</td></tr>
<tr><td></td><td></td><td></td><td>....|....|....|....|</td></tr>
<tr><td></td><td></td><td></td><td>GATTGTGTGCGTACTGCTGC</td></tr>
<tr><td>1</td><td>16928</td><td>99.419</td><td>....................</td></tr>
<tr><td>2</td><td>6</td><td>0.035</td><td>.............t......</td></tr>
<tr><td>3</td><td>2</td><td>0.012</td><td>........t...........</td></tr>
<tr><td>4</td><td>1</td><td>0.006</td><td>................g...</td></tr>
<tr><td>outgroup1</td><td>86</td><td>0.505</td><td></td></tr>
<tr><td>outgroup2</td><td>3</td><td>0.018</td><td></td></tr>
<tr><td>excluded</td><td>1</td><td>0.006</td><td></td></tr>
</table>

Niu-E

<table>
<tr><th>Group Number</th><th>Variant Count</th><th>Frequency %</th><th>Forward</th></tr>
<tr><td></td><td></td><td></td><td>10 20</td></tr>
<tr><td></td><td></td><td></td><td>....|....|....|....|</td></tr>
<tr><td></td><td></td><td></td><td>TTCTTGCTTTCGTGGTATTC</td></tr>
<tr><td>1</td><td>16926</td><td>99.407</td><td>....................</td></tr>
<tr><td>2</td><td>9</td><td>0.053</td><td>................t...</td></tr>
<tr><td>3</td><td>7</td><td>0.041</td><td>...........a........</td></tr>
<tr><td>4</td><td>3</td><td>0.018</td><td>..........t.........</td></tr>
<tr><td>5</td><td>1</td><td>0.006</td><td>.....t..............</td></tr>
<tr><td>6</td><td>1</td><td>0.006</td><td>......t.............</td></tr>
<tr><td>7</td><td>1</td><td>0.006</td><td>..t.................</td></tr>
<tr><td>8</td><td>1</td><td>0.006</td><td>...........t........</td></tr>
<tr><td>outgroup1</td><td>77</td><td>0.452</td><td></td></tr>
<tr><td>outgroup2</td><td>1</td><td>0.006</td><td></td></tr>
</table>

<table>
<tr><th>Group Number</th><th>Variant Count</th><th>Frequency %</th><th>Probe</th></tr>
<tr><td></td><td></td><td></td><td>10 20 30</td></tr>
<tr><td></td><td></td><td></td><td>....|....|....|....|....|....|</td></tr>
<tr><td></td><td></td><td></td><td>GTTACACTAGCCATCCTTACTGCGCTTCGA</td></tr>
<tr><td>1</td><td>16921</td><td>99.377</td><td>..............................</td></tr>
<tr><td>2</td><td>17</td><td>0.100</td><td>...........t..................</td></tr>
<tr><td>3</td><td>3</td><td>0.018</td><td>....t.........................</td></tr>
<tr><td>4</td><td>1</td><td>0.006</td><td>......................t.......</td></tr>
<tr><td>5</td><td>1</td><td>0.006</td><td>.........................g....</td></tr>
<tr><td>6</td><td>1</td><td>0.006</td><td>.........................a....</td></tr>
<tr><td>7</td><td>1</td><td>0.006</td><td>........................t.....</td></tr>
<tr><td>outgroup1</td><td>79</td><td>0.464</td><td></td></tr>
<tr><td>outgroup2</td><td>3</td><td>0.018</td><td></td></tr>
</table>

<table>
<tr><th>Group Number</th><th>Variant Count</th><th>Frequency %</th><th>Reverse</th></tr>
<tr><td></td><td></td><td></td><td>10 20</td></tr>
<tr><td></td><td></td><td></td><td>....|....|....|....|</td></tr>
<tr><td></td><td></td><td></td><td>GCTGCAATATTGTTAACGTG</td></tr>
<tr><td>1</td><td>16911</td><td>99.319</td><td>....................</td></tr>
<tr><td>2</td><td>2</td><td>0.012</td><td>.................t..</td></tr>
<tr><td>3</td><td>1</td><td>0.006</td><td>.g..................</td></tr>
<tr><td>4</td><td>1</td><td>0.006</td><td>................t...</td></tr>
<tr><td>5</td><td>1</td><td>0.006</td><td>........t...........</td></tr>
<tr><td>outgroup1</td><td>109</td><td>0.640</td><td></td></tr>
<tr><td>excluded</td><td>2</td><td>0.012</td><td></td></tr>
</table>

NIH-TH_N

<table>
<tr><th>Group Number</th><th>Variant Count</th><th>Frequency %</th><th>Forward
10 20</th></tr>
<tr><td></td><td></td><td></td><td>....|....|....|....|</td></tr>
<tr><td></td><td></td><td></td><td>CGTTTGGTGGACCCTCAGAT</td></tr>
<tr><td>1</td><td>16893</td><td>99.213</td><td>....................</td></tr>
<tr><td>2</td><td>58</td><td>0.341</td><td>..................g.</td></tr>
<tr><td>3</td><td>7</td><td>0.041</td><td>...........t........</td></tr>
<tr><td>4</td><td>6</td><td>0.035</td><td>.........t..........</td></tr>
<tr><td>5</td><td>2</td><td>0.012</td><td>.................t..</td></tr>
<tr><td>6</td><td>2</td><td>0.012</td><td>.t..................</td></tr>
<tr><td>7</td><td>2</td><td>0.012</td><td>............t.......</td></tr>
<tr><td>8</td><td>2</td><td>0.012</td><td>t...................</td></tr>
<tr><td>9</td><td>1</td><td>0.006</td><td>......t.............</td></tr>
<tr><td>10</td><td>1</td><td>0.006</td><td>........c...........</td></tr>
<tr><td>11</td><td>1</td><td>0.006</td><td>.....a..............</td></tr>
<tr><td>outgroup1</td><td>51</td><td>0.300</td><td></td></tr>
<tr><td>outgroup2</td><td>1</td><td>0.006</td><td></td></tr>
</table>

<table>
<tr><th>Group Number</th><th>Variant Count</th><th>Frequency %</th><th>Probe
10</th></tr>
<tr><td></td><td></td><td></td><td>....|....|....|.</td></tr>
<tr><td></td><td></td><td></td><td>CAACTGGCAGTAACCA</td></tr>
<tr><td>1</td><td>16946</td><td>99.524</td><td>................</td></tr>
<tr><td>2</td><td>25</td><td>0.147</td><td>...a............</td></tr>
<tr><td>3</td><td>6</td><td>0.035</td><td>..............g.</td></tr>
<tr><td>4</td><td>4</td><td>0.023</td><td>...t............</td></tr>
<tr><td>5</td><td>1</td><td>0.006</td><td>...............g</td></tr>
<tr><td>6</td><td>1</td><td>0.006</td><td>...........t....</td></tr>
<tr><td>7</td><td>1</td><td>0.006</td><td>.......t........</td></tr>
<tr><td>8</td><td>1</td><td>0.006</td><td>......t.........</td></tr>
<tr><td>outgroup1</td><td>42</td><td>0.247</td><td></td></tr>
</table>

<table>
<tr><th>Group Number</th><th>Variant Count</th><th>Frequency %</th><th>Reverse
10</th></tr>
<tr><td></td><td></td><td></td><td>....|....|....|....</td></tr>
<tr><td></td><td></td><td></td><td>AATGGAGAACGCAGTGGGG</td></tr>
<tr><td>1</td><td>16938</td><td>99.477</td><td>...................</td></tr>
<tr><td>2</td><td>8</td><td>0.047</td><td>.............t.....</td></tr>
<tr><td>3</td><td>6</td><td>0.035</td><td>................a..</td></tr>
<tr><td>4</td><td>5</td><td>0.029</td><td>...............t...</td></tr>
<tr><td>5</td><td>4</td><td>0.023</td><td>..........a........</td></tr>
<tr><td>6</td><td>2</td><td>0.012</td><td>................t..</td></tr>
<tr><td>7</td><td>2</td><td>0.012</td><td>....t..............</td></tr>
<tr><td>8</td><td>2</td><td>0.012</td><td>..................t</td></tr>
<tr><td>9</td><td>2</td><td>0.012</td><td>...............tt..</td></tr>
<tr><td>10</td><td>1</td><td>0.006</td><td>...c...............</td></tr>
<tr><td>11</td><td>1</td><td>0.006</td><td>...........t.......</td></tr>
<tr><td>12</td><td>1</td><td>0.006</td><td>.g.................</td></tr>
<tr><td>13</td><td>1</td><td>0.006</td><td>.........t.........</td></tr>
<tr><td>14</td><td>1</td><td>0.006</td><td>..................a</td></tr>
<tr><td>15</td><td>1</td><td>0.006</td><td>.c.................</td></tr>
<tr><td>outgroup1</td><td>52</td><td>0.305</td><td></td></tr>
</table>

US-CDC-N-2

<table>
<tr><th>Group Number</th><th>Variant Count</th><th>Frequency %</th><th>Forward
10 20</th></tr>
<tr><td></td><td></td><td></td><td>....|....|....|....|</td></tr>
<tr><td></td><td></td><td></td><td>TTACAAACATTGGCCGCAAA</td></tr>
<tr><td>1</td><td>16647</td><td>97.768</td><td>....................</td></tr>
<tr><td>2</td><td>25</td><td>0.147</td><td>...t................</td></tr>
<tr><td>3</td><td>8</td><td>0.047</td><td>.......t............</td></tr>
<tr><td>4</td><td>4</td><td>0.023</td><td>...............t....</td></tr>
<tr><td>5</td><td>2</td><td>0.012</td><td>.c..................</td></tr>
<tr><td>6</td><td>1</td><td>0.006</td><td>.........aa.........</td></tr>
<tr><td>7</td><td>1</td><td>0.006</td><td>............t.......</td></tr>
<tr><td>outgroup1</td><td>339</td><td>1.991</td><td></td></tr>
</table>

<table>
<tr><th>Group Number</th><th>Variant Count</th><th>Frequency %</th><th>Probe
10 20</th></tr>
<tr><td></td><td></td><td></td><td>....|....|....|....|...</td></tr>
<tr><td></td><td></td><td></td><td>ACAATTTGCCCCCAGCGCTTCAG</td></tr>
<tr><td>1</td><td>16605</td><td>97.522</td><td>.......................</td></tr>
<tr><td>2</td><td>34</td><td>0.200</td><td>t......................</td></tr>
<tr><td>3</td><td>26</td><td>0.153</td><td>............t..........</td></tr>
<tr><td>4</td><td>4</td><td>0.023</td><td>...............t.......</td></tr>
<tr><td>5</td><td>3</td><td>0.018</td><td>......c................</td></tr>
<tr><td>6</td><td>1</td><td>0.006</td><td>..................c....</td></tr>
<tr><td>7</td><td>1</td><td>0.006</td><td>..............at.t.....</td></tr>
<tr><td>8</td><td>1</td><td>0.006</td><td>...........t...........</td></tr>
<tr><td>9</td><td>1</td><td>0.006</td><td>.......t...............</td></tr>
<tr><td>outgroup1</td><td>351</td><td>2.061</td><td></td></tr>
</table>

<table>
<tr><th>Group Number</th><th>Variant Count</th><th>Frequency %</th><th>Reverse
10</th></tr>
<tr><td></td><td></td><td></td><td>....|....|....|...</td></tr>
<tr><td></td><td></td><td></td><td>TTCTTCGGAATGTCGCGC</td></tr>
<tr><td>1</td><td>16677</td><td>97.944</td><td>..................</td></tr>
<tr><td>2</td><td>7</td><td>0.041</td><td>.....t............</td></tr>
<tr><td>3</td><td>3</td><td>0.018</td><td>..............t...</td></tr>
<tr><td>4</td><td>2</td><td>0.012</td><td>..............a...</td></tr>
<tr><td>5</td><td>1</td><td>0.006</td><td>.................t</td></tr>
<tr><td>6</td><td>1</td><td>0.006</td><td>...............a..</td></tr>
<tr><td>7</td><td>1</td><td>0.006</td><td>................a.</td></tr>
<tr><td>8</td><td>1</td><td>0.006</td><td>.......a..........</td></tr>
<tr><td>outgroup1</td><td>334</td><td>1.962</td><td></td></tr>
</table>

Young-N

Group Number	Variant Count	Frequency %	Forward
			10 20
			....|....|....|....|..
			CTCAGTCCAAGATGGTATTTCT
1	16953	99.565	....
2	5	0.029	....................t.
3	1	0.006	...................c..
4	1	0.006	..............c.......
outgroup1	67	0.393	

Group Number	Variant Count	Frequency %	Probe
			10 20
			....|....|....|....|...
			ACCTAGGAACTGGCCCAGAAGCT
1	16927	99.413	.............G.........
2	21	0.123	.............g.......t.
3	11	0.065	.............g......t..
4	4	0.023	.............a.........
5	1	0.006	.............g...t.....
6	1	0.006	.............g.....g...
7	1	0.006	.......g.....g.........
8	1	0.006	.t...........g.........
9	1	0.006	.............gt........
10	1	0.006	..a..........g.........
outgroup1	58	0.341	

Group Number	Variant Count	Frequency %	Reverse
			10
			....|....|....|...
			GGACTTCCCTATGGTGCT
1	16949	99.542	....
2	15	0.088	.......t..........
3	6	0.035	c
4	2	0.012	........t.........
5	1	0.006	....a.............
6	1	0.006	...............a..
7	1	0.006	.....c............
outgroup1	51	0.300	
outgroup2	1	0.006	

Corman-N

Group Number	Variant Count	Frequency %	Forward
			10
			....|....|....|....
			CACATTGGCACCCGCAATC
1	16954	99.571	....
2	16	0.094	..t................
3	9	0.053	..............t....
4	3	0.018	t..................
5	2	0.012	..................t
6	2	0.012	...........t.......
7	2	0.012	...t...............
8	1	0.006	..........t........
outgroup1	38	0.223	

Group Number	Variant Count	Frequency %	Probe
			10 20
			....|....|....|....|....|
			ACTTCCTCAAGGAACAACATTGCCA
1	16930	99.430	....
2	4	0.023	.................t.......
3	3	0.018	.......a.................
4	3	0.018	.....t...................
5	2	0.012	.t.......................
6	2	0.012	......................t..
7	2	0.012	.....................t...
8	2	0.012	.......................a.
9	2	0.012	............g............
10	1	0.006	...............t.........
11	1	0.006	.........g...............
outgroup1	75	0.440	

Group Number	Variant Count	Frequency %	Reverse
			10 20
			....|....|....|....|
			CAAGCCTCTTCTCGTTCCTC
1	16863	99.037	....
2	32	0.188	.......a............
3	19	0.112	............t.......
4	6	0.035	...................t
5	5	0.029	.............t......
6	2	0.012	................t...
7	1	0.006	g...................
8	1	0.006	..........g.........
9	1	0.006	.............a......
10	1	0.006	....t...............
11	1	0.006	................a...
12	1	0.006	...a................
13	1	0.006	......a.............
14	1	0.006	......c.............
outgroup1	92	0.540	

Won-N

Group Number	Variant Count	Frequency %	Forward
			10 20
			....|....|....|....|
			CAATGCTGCAATCGTGCTAC
1	16953	99.565	....
2	27	0.159	.......t............
3	6	0.035	.....t..............
4	3	0.018	............t.......
5	1	0.006	.t..................
6	1	0.006	.....a..............
7	1	0.006	t...................
8	1	0.006	...............t....
9	1	0.006	...........c........
outgroup1	33	0.194	

Group Number	Variant Count	Frequency %	Reverse
			10 20
			....|....|....|....|
			CCTCATCACGTAGTCGCAAC
1	16788	98.596	....
2	61	0.358	......t.............
3	31	0.182	............t.......
4	16	0.094	.....c..............
5	14	0.082	...................t
6	6	0.035	...............t....
7	6	0.035	...t................
8	5	0.029	..............t.....
9	4	0.023	...........g........
10	2	0.012	t...................
11	2	0.012	...................g
12	2	0.012	................t...
13	1	0.006	a...................
14	1	0.006	..............a.....
15	1	0.006	..................g.
16	1	0.006	..............g.....
17	1	0.006	....g...............
outgroup1	84	0.493	
outgroup2	1	0.006	

NIID-JP-N

Group Number	Variant Count	Frequency %	Forward
			10 20
			....|....|....|....|
			AAATTTTGGGGACCAGGAAC
1	16658	97.833	....
2	60	0.352	...................t
3	3	0.018	...............t....
4	2	0.012	..........a.........
5	1	0.006	...............c....
6	1	0.006	.........a..........
7	1	0.006	.c..................
outgroup1	301	1.768	

Group Number	Variant Count	Frequency %	Probe
			10 20
			....|....|....|....|
			ATGTCGCGCATTGGCATGGA
1	16679	97.956	....
2	3	0.018	.....t..............
3	3	0.018	.................a..
4	2	0.012	.....a..............
5	2	0.012	.................t..
6	1	0.006	........t...........
7	1	0.006	.........g..........
8	1	0.006	.............a......
9	1	0.006	......a.............
10	1	0.006	..................t.
11	1	0.006	................c...
12	1	0.006	.......a............
13	1	0.006	............c.......
14	1	0.006	...............g....
outgroup1	329	1.932	

Group Number	Variant Count	Frequency %	Reverse
			10 20
			....|....|....|....|
			GTTGACCTACACAGCTGCCA
1	16687	98.003	..............G.....
2	10	0.059	...........t..g.....
3	4	0.023	.........t....g.....
4	4	0.023	......t.......g.....
5	3	0.018	...t..........g.....
6	2	0.012	.....t........g.....
7	2	0.012	..............g...t.
8	2	0.012	..............g..t..
9	1	0.006	..............g....t
10	1	0.006	..........t...g.....
11	1	0.006	..............gc....
12	1	0.006	.c............g.....
outgroup1	309	1.815	

HKU-N

Group Number	Variant Count	Frequency %	Forward
			10 20
			....|....|....|....|..
			TAATCAGACAAGGAACTGATTA
1	16667	97.886	....
2	41	0.241	...c..................
3	3	0.018	....t.................
4	2	0.012	....................c.
5	1	0.006	.........t............
6	1	0.006	a.....................
7	1	0.006	...............t......
8	1	0.006	.................t....
9	1	0.006	................c.....
outgroup1	309	1.815	

Group Number	Variant Count	Frequency %	Probe *
			10 20
			....|....|....|....|
			CCGCAAATTGCACAATTTGC
1	16637	97.710	....
2	34	0.200	...........t........
3	4	0.023	..t.................
4	3	0.018	.................c..
5	1	0.006	..........t.........
6	1	0.006	..................t.
outgroup1	347	2.038	

Group Number	Variant Count	Frequency %	Reverse
			10
			....|....|....|....
			CATGGAAGTCACACCTTCG
1	16668	97.892	....
2	13	0.076	.................t.
3	10	0.059	..................t
4	4	0.023	..............t....
5	3	0.018	...a...............
6	2	0.012	...t...............
7	2	0.012	...........t.......
8	1	0.006	.............t.....
9	1	0.006	....t..............
10	1	0.006	..c................
11	1	0.006	........c..........
12	1	0.006	.g.................
outgroup1	320	1.879	

* The binding region of probe is reverse complemented.

Chan-N

Group Number	Variant Count	Frequency %	Forward
			10
			....|....|....|...
			GCGTTCTTCGGAATGTCG
1	16665	97.874	....
2	8	0.047	..t...............
3	7	0.041	........t.........
4	3	0.018	.................t
5	2	0.012	.t................
6	2	0.012	.................a
7	1	0.006	..c...............
8	1	0.006	..........a.......
outgroup1	338	1.985	

Group Number	Variant Count	Frequency %	Probe
			10 20
			....|....|....|....|..
			AACGTGGTTGACCTACACAGST
			S=G/C
1	16680	97.962	....................G.
2	10	0.059	.................t..g.
3	4	0.023	...t................g.
4	4	0.023	...............t....g.
5	4	0.023	............t.......g.
6	3	0.018	.........t..........g.
7	3	0.018	..t.................g.
8	2	0.012	...........t........g.
9	2	0.012	.....t..............g.
10	1	0.006	...a................g.
11	1	0.006	................t...g.
12	1	0.006	....................gc
13	1	0.006	.......c............g.
outgroup1	310	1.821	
outgroup2	1	0.006	

Group Number	Variant Count	Frequency %	Reverse
			10 20
			....|....|....|....|...
			CAAATTGGATGACAAAGATCCAA
1	16674	97.927	....
2	19	0.112	...................t...
3	10	0.059	t......................
4	6	0.035	............t..........
5	5	0.029	.......a...............
6	3	0.018	....................t..
7	2	0.012	......t................
8	1	0.006	.................t.....
9	1	0.006	.......t...............
10	1	0.006	........g..............
11	1	0.006	......a................
outgroup1	304	1.785	
